# Use of Follicular Output Rate to Predict Intracytoplasmic Sperm Injection Outcome

**DOI:** 10.22074/ijfs.2016.4906

**Published:** 2016-06-01

**Authors:** Rehana Rehman, Rozina Mustafa, Mukhtiar Baig, Sara Arif, Muhammad Faisal Hashmi

**Affiliations:** 1 Department of Biological and Biomedical Sciences, The Aga Khan University, Karachi, Pakistan; 2 Department of Obstetrics and Gynecology, United Medical and Dental College, Karachi, Pakistan; 3 Department of Biochemistry, Faculty of Medicine, Rabigh, King Abdul Aziz University, Jeddah, Saudi Arabia; 4 House Officer Civil Hospital, Karachi, Pakistan; 5 House Officer, Services Hospital, Lahore, Pakistan

**Keywords:** Intracytoplasmic Sperm Injection, Assisted Reproductive Techniques, Infer- tility, Ovarian Follicles, Follicular Output Rate

## Abstract

**Background:**

The measurement of follicular output rate (FORT) has been proposed as a good indicator for evaluating follicular response to the exogenous recombinant folliclestimulating hormone (rFSH). This places FORT as a promising qualitative marker for ovarian function. The objective of the study was to determine FORT as a predictor of oocyte competence, embryo quality and clinical pregnancy after intracytoplasmic sperm injection (ICSI).

**Materials and Methods:**

This prospective study was carried out on a group of infer- tile females (n=282) at Islamabad Clinic Serving Infertile Couples, Islamabad, Pakistan, from June 2010 till August 2013. Downregulated females were stimulated in injection gonadotropins and on ovulation induction day, pre-ovulatory follicle count (PFC) was determined using transvaginal ultrasound scan (TVUS), and FORT was determined as a ratio of PFC to antral follicle count (AFC)×100. Group I consisted of females with a negative pregnancy test, while group II had a positive pregnancy test that was confirmed with the appearance of fetal cardiac activity. Linear regression analyses of categorical variables of clinical pregnancy along with other independent variables, including FORT, were performed using SPSS version 15.0.

**Results:**

Pregnancy occurred in 101/282 women who were tested, recording a clinical pregnancy rate of about 35.8%. FORT values were higher in group II as compared to group I females (P=0.0001). In multiple regression analysis, 97.7, 87.1, 78.2, and 83.4% variations were explained based on the number of retrieved oocytes per patients, number of metaphase II oocytes retrieved, number of fertilized oocytes, and number of cleaved embryos, respectively, indicating FORT as an independent predictor.

**Conclusion:**

FORT is a predictor of oocyte competence in terms of a number of retrieved, mature and fertilized oocytes. It also gives information about the number of cleaved embryos and clinical pregnancy rate.

## Introduction

The prevalence of infertility in Pakistan is 21.9%, which accounts for approximately 3.5 to 3.9% cases of primary infertility and 18.0 to 18.4% of secondary infertility (1). The assisted reproductive clinics offer scientific assistance to infertile couples for the commencement of pregnancy preparation. Assisted reproductive techniques include *in vitro* fertilization (IVF) and intracytoplasmic sperm injection (ICSI) for the treatment of infertile couples (2,3). 

In ICSI, after the down-regulation of ovaries, controlled ovarian hyperstimulation (COH) is followed by ovulation induction (OI) and oocyte pickup, after which a single sperm is introduced in the ooplasm, placed under a microscope. The procedure has shown a greater success rate as compared to IVF. Ovarian stimulation is a key procedure necessary to achieve success in assisted reproductive techniques. Stimulation is achieved by the administration of exogenous gonadotropins in the form of recombinant follicle stimulating hormone (rFSH) in order to increase the follicular recruitment and oocyte yield. Confirmation of responsiveness of ovarian reservoir to FSH in terms of the development of the antral follicles is so far a challenge for reproductive endocrinologists at clinics (1,4). The appropriate response of antral follicles to FSH and a high-quality oocyte may result in a positive impact on outcomes of IVF and ICSI. Poor response to COH results in retrieval of few oocytes with reduced number of embryos available for transfer, leading to a decrease in pregnancy rates (2). 

The endocrinologists, thus, devised various methods to assess the ovarian reservoir and the expected responsiveness. Antral follicle count (AFC) is one of the noninvasive methods used for the assessment of the sensitivity of antral follicles to FSH (5). AFC represents the number of remaining primordial pool which corresponds to the number of oocytes retrieved; however, it does not influence the number of oocytes, embryo quality and the outcome of ICSI (6). The number of pre-ovulatory follicle count (PFC) obtained at the end of COH is estimated to be the best indicator of the number of retrieved oocytes (2,3,7). 

However, it also includes the number of small antral follicles available before treatment (8,9). It means that follicular output rate (FORT) determines follicular response to exogenous rFSH by the ratio of pre-ovulatory follicles to the existing pool of small antral follicles. The index has been investigated as an indicator of existing ovarian reservoir in response to stimulation and oocyte competence by a number of researchers (1,4,5). In addition to assessing ovarian function, we designed this study in order to identify the value of FORT for the prediction of oocyte competence, embryo quality and the likelihood of clinical pregnancy after ICSI. 

## Materials and Methods

This prospective study was carried out at Islamabad Clinic Serving Infertile Couples Islamabad, Pakistan, between June 2010 and August 2013 after the ethical approval was obtained from the Ethical Review Board of Islamabad Clinic Serving Infertile Couples, Islamabad, Pakistan. The study was conducted in accordance with the principles of the Declaration of Helsinki, and an informed written consent was taken from all study participants. The women (n=282) undergoing ICSI were included in this study with the following criteria: infertility duration of more than 2 years, age range of 21-40 years, regular menstrual cycle, normal morphological ovaries, body mass index (BMI) of 18-35 kg/ m ^2^, and serum FSH levels lower than 8 IU/ml. The exclusion criteria were as follows: polycystic ovary syndrome (PCOS), uterine fibroids, down-regulation with short gonadotropin-releasing hormone (GnRH)-agonist or -antagonist protocol, sperm retrieval by testicular biopsy, and use of IVF cycles. 

### Treatment protocol

The treatment protocol of females was carried out as mentioned by Rehman et al. (3). Total number of AFC with a mean diameter of 3-8 mm was determined using a transvaginal ultrasound scan (TVUS) with a 7.5 MHz probe (Aloka Co., Japan). 

COH was carried out using subcutaneous administration of 50 IU/day rFSH (Puregon, NV Organon, The Netherlands). The initiation dose was calculated based on the of the age of the subject, basal serum FSH concentrations, AFC and BMI (6). Dose adjustment was done by follicular tracking with TVUS, commenced from the fifth day of COH on alternate days for assessment of follicular response and measurement of endometrial thickness. During the last days of COH, patients had to visit the assisted reproductive clinics daily for the appropriate time of OI using human chorionic gonadotropin (hCG) injection (1). On OI day, PFC was calculated as the total number of follicles with a mean diameter of 16-22 mm in both ovaries using TVUS. 

The FORT was calculated as the ratio of PFC on OI day ×100/AFC (10). Approximately 37 hours after hCG injection, oocyte pick-up was performed under general anesthesia using a vaginal ultrasound probe, after which luteal support was provided with the progesterone vaginal pessaries (Cyclogest 400 mg, Actavis Co., UK) twice a day. Transfer of blastocysts was performed seven days after hCG injection using Edwards-Wallace embryo replacement catheter (SIMS Portex Ltd., UK) under ultrasound guidance. Two blood samples were taken on days of hCG injection and blastocyst transfer for estimation of peak and mid-luteal levels of estradiol (E_2_) and progesterone using enzyme linked immuno sorbent assay (ELISA). Serum E_2_and progesterone as well as ELISA kits were provided by MP Biomedicals, USA and BioSource, Belgium, respectively. 

### Outcome parameters

The marker for detection of pregnancy outcome was beta-hCG (β-hCG) levels that were measured 14 days after egg collection. Clinical pregnancy was identified by the appearance of a gestational sac with cardiac activity using TVUS. Therefore, the participants were divided into the following groups: group I (n=181) including non-pregnant women with β-hCG<25 mIU/ml, and group II (n=101) including pregnant women with β-hCG>25 mIU/ml and the presence of cardiac activity confirmed by TVUS. Pregnancy rate was calculated by the presence of an intrauterine gestational sac observed by TVUS per number of patients in the cycle (5,7). 

### Statistical analysis

Data was analyzed by Statistical Package for Social Science software (SPSS, SPSS Inc., USA) version 15.0. The stepwise backward method was used to estimate the best model using FORT as a predictor that was adjusted with other clinical risk factors affecting clinical pregnancy outcome, including female age, BMI, FSH, duration of infertility, the length of stimulation, AFC, and PFC. A variable is entered into the model if the significance level of its F value is less than 0.05 and removed if the significance level is greater than 0.10. Four different models were developed showing that FORT gave significant effect on the number of oocytes/patient, the number of metaphase II oocytes retrieved, the number of cleaved embryos and number of fertilized oocytes. In regression analysis, R-squared (R ^2^, coefficient of determination) described variation in dependent variable with respect to independent variable(s), while Spearman’s Rank Correlation was used to investigate the correlation of clinical pregnancy with different parameters. A P value less than 0.05 was considered to be statistically significant. 

### Results

Baseline characteristics of study participants are given in Table 1. Pregnancy occurred in 101/282 women who were tested, recording a clinical pregnancy rate of about 35.8%. Table 2 explains the significant difference regarding FORT values in group I (49.31 ± 12.83) and II (64.17 ± 8.96). 

**Table 1:** Baseline characteristics of the infertile subjects


Parameters	Mean	SD

Female age (Y)	32.18	4.72
Duration of infertility (Y)	7.46	4.01
Length of stimulation (days)	14.16	0.96
AFC	13.07	2.56
PFC	8.31	1.71
FORT	64.09	8.97
No. of oocytes/patient	8.21	1.53
No. of metaphase II oocytes retrieved	8.03	1.45
No. of fertilized oocytes	6.65	1.07
No. of cleaved embryos	6.56	1.04
Peak progesterone (ng/ml)	0.85	0.45
Mid-luteal progesterone (ng/ml)	89.17	32.84
Peak E_2_ (pg/ml)	2529.06	193.99
Mid-luteal E_2_ (pg/ml)	1109.46	136.29
Endometrial thicknessa (mm)	1.74	0.44


Values are presented as mean ± SD. AFC; Antral follicle count, PFC; Pre- ovulatory follicle count, FORT; Follicular output rate, and E_2_ ; Estradiol.

**Table 2:** Comparison of the parameters between group I (non-pregnant participants) and group II (pregnant participants)


Parameters	Non-pregnant participants n=181	Pregnant participants n=101	P value

Female age (Y)	32.11 ± 4.61	32.107 ± 4.74	0.952
Duration of infertility (Y)	6.94 ± 3.78	7.40 ± 4.02	0.409
Length of stimulation (days)	14.43 ± 0.96	14.15 ± 0.95	- 0.454
Dose of rFSH/day	3.89 ± 1.43	4.29 ± 2.96	<0.001
AFC	15.57 ± 2.51	13.04 ± 2.55	0.559
PFC	7.52 ± 1.90	8.30 ± 1.69	0.364
FORT	49.31 ± 12.83	64.17 ± 8.96	<0.001
No. of cleaved embryos	5.45 ± 1.53	6.56 ± 1.03	<0.001
Endometrial thickness (mm)	1.26 ± 0.44	1.74 ± 0.43	0.819
No. of oocytes/ patient	7.40 ± 1.68	8.20 ± 1.51	0.296
No. of mature oocytes/patient	6.61 ± 2.03	8.02 ± 1.43	0.004
No. of fertilized oocytes	5.54 ± 1.64	6.65 ± 1.06	<0.001
Peak E_2_(pg/ml)	2207.97 ± 283.73	2529.06 ± 193.35	<0.01
Mid-luteal E_2_ (pg/ml)	899.49 ± 122.05	1109.46 ± 135.83	<0.01
Peak progesterone (ng/ml)	1.96 ± 0.67	1.12 ± 0.59	<0.01
Mid-luteal progesterone ng/ml	192.44 ± 20.34	116.51 ± 48.60	<0.01


Values are presented as mean ± SD. rFSH; Recombinant folicle stimulating hormone, AFC; Antral follicle count, PFC; Pre ovula- tory follicle count, FORT; Follicular output rate, and E_2_ ; Estradiol.

**Table 3:** Comparison of the parameters for clinical pregnancy outcome using Spearman rank correlation


Predictors	Clinical pregnancy r(P value)

Female age (Y)	0.001(0.981)
Duration of infertility (Y)	0.052(0.380)
Length of stimulation (days)	-0.140^*^(0.018)
AFC	-0.412^*^(<0.01)
PFC	0.265^*^(<0.01)
FORT	0.532^*^(<0.01)
No. of cleaved embryos	0.365^*^(<0.01)
Peak progesterone (ng/ml)	-0.652^*^(<0.01)
Mid luteal progesterone (ng/ml)	-0.802^*^(<0.01)
Peak E_2_ (pg/ml)	0.640^*^(<0.01)
Mid luteal E_2_ (pg/ml)	0.679^*^(<0.01)
Endometrial thickness	0.628^*^(<0.01)


AFC; Antral follicle count, PFC; Pre-ovulatory follicle count, FORT; Follicular output rate, E_2_ ; Estradiol, *; Correlation with P<0.05 considered as significant, and r; Spearman rank correlation.

**Table 4:** Regression models with FORT as an independent predictor


Regression models	Independent predictor	Beta coefficient	SE	Adjusted R^2^	P value

No. of oocytes/patients^a^	FORT	0.135	± 0.003	97.7%	<0.01^*^
No. of metaphase II oocytes retrieved^b^	FORT	0.128	± 0.006	87.1%	<0.01^*^
No. of fertilized oocytes^c^	FORT	0.089	± 0.006	78.2%	<0.01^*^
No. of cleaved embryos^d^	FORT	-0.025	± 0.01	83.4%	0.019^*^


FORT; Follicular output rate, R^2^ ; Coefficient of determination, AFC; Antral follicle count, FSH; Follicle stimulating hormone, ^a^; Adjusted with length of stimulation and AFC, ^b,c^; Adjusted with FSH and AFC, ^d^; Adjusted with AFC and number of metaphase II oocytes retrieved and ^*^; P<0.05 was considered as significant.

It indicates that the length of stimulation, AFC (r=0.412, P<0.01), as well as peak (r=-0.640, P<0.01) and mid-luteal progesterone levels (r=-0.802, P<0.01) showed a significant negative association with clinical pregnancy outcome. However, FORT (r=0.532, P<0.001), PFC (r=0.265, P<0.01), number of cleaved embryos (r=0.365, P<0.01), peak (r=0.640, P<0.01) and mid-luteal (r=0.679, P<0.01), E_2_ (r=0.640, P<0.01) levels, as well as endometrial thickness (r=670, P<0.01) showed a significant positive correlation with clinical pregnancy outcome (Table 3). Linear regression analysis of FORT as an independent predictor described variations of 97.7, 87.1, 78.2 and 83.4% for the number of retrieved oocytes per patients, number of metaphase II oocytes retrieved, the number of fertilized oocytes, and the number of cleaved embryos, respectively (Table 4). Furthermore, table 4 shows an increase in FORT value by one unit increased the mean number of oocytes/patients (β coefficient: 0.135), while the model was adjusted for length of stimulation and AFC with an adjusted R 2 value of 97.7%. The model describes that an increase in FORT value by one unit increased the mean number of metaphase II oocytes retrieved (β coefficient: 0.128), while the model was adjusted for FSH and AFC with an adjusted R 2 value of 87.1%. The model also describes that an increase in FORT value by one unit will give a positive impact on the mean number of oocytes fertilized (β coefficient: 0.089), while the model was adjusted for FSH and AFC with an adjusted R_2_value of 78.2 %. However, the model suggested that one unit increase in FORT value decreased the mean number of cleaved embryos (β coefficient:-0.025), while it was adjusted for AFC and number of metaphase II oocytes retrieved (Table 4).

### Discussion

The successful outcome in assisted reproductive clinics depends on embryos and endometrial qualities. The quality of embryos depends on number and quality of oocytes, which in turn is considered as a measure of antral follicles responsiveness to FSH; however, no pointer can predict both ovarian response and oocyte competence. FORT, in this regard, can be used as an innovative marker to predict ovarian response and oocyte competence, leading to increase the chances of clinical pregnancy (8). 

AFC as a non-invasive method of assessment plays a key role in the prediction of ovarian response and provides a valuable insight into the sensitivity of antral follicles to FSH (5). In this study, AFC showed significant negative association with the pregnancy outcome. Similar to our findings, a study reported that AFC represented the actual ovarian reserve and were highly correlated with the number of oocytes retrieved (6). Furthermore, other studies concluded that AFC cannot predict the oocyte/embryo quality or IVF/ ICSI outcome and showed better FORT results in subjects with decreased AFC (4,11). A study declared that AFC obtained at the end of stimulation is the best indicator of the number of retrieved oocytes and good clinical outcome (7). However, a contrasting observation was noted in another study where pre-ovulatory follicles were not observed as an appropriate reflector of antral follicle sensitivity to FSH (12). The conflict can thus be resolved by the use of FORT index, which can reflect ovarian follicular competence in response to stimulation (4,5). 

High FORT showed a higher rate of clinical pregnancies that can be explained on the basis of its association with basal, peak and mid-luteal E_2_concentrations (8). The role of high FORT in increasing peak E_2_concentrations can be explained by increased retrieval, maturity and fertilization of oocytes, which potentially lead to an increase in clinical pregnancy rate (10,13,14). Enhanced midluteal E_2_concentration in females with high FORT increases the chance of pregnancy, which is also supported by other studies (3,8,15). 

The exact role of progesterone in determining the success of IVF/ ICSI is a subject of debate. Our results showed that females with high FORT had low peak progesterone levels, which is similar to another study in which pregnancy rate was inversely related to serum progesterone levels (16). However, in contrast to these results, a study proved that progesterone enhances uterine receptivity by the maturation of mast cells that produce cytokines and growth factors (17,18). Hofmann et al. (19) observed no significant difference in pregnancy rate in patients undergoing IVF/embryo transfer with high or low progesterone concentrations on the day of hCG administration and in patients receiving oocytes donated from women with high or low progesterone concentrations. The role of FORT in the prediction of clinical pregnancy can thus be emphasized on the basis of its effect on hormones of implantation. 

It seems that FORT is a qualitative indicator of ovarian follicles competence and has a significant correlation with the clinical pregnancy outcome. 

Our results showed that it has positive effects on a number of oocytes/patient, the number of metaphase II oocytes retrieved, the number of cleaved embryos and number of fertilized oocytes. Similar to other two studies that reflected the rates of good quality embryos and embryo implantations, clinical pregnancies increased dramatically in accordance with FORT values (4,20). Besides the importance of the impact of FORT on clinical pregnancy, it was a unicentric study in which different sonologists determined AFC and PFC which must be standardized for uniformity . The variation in marking AFC and PFC by different sonographers is unlikely to be ruled out. 

## Conclusion

FORT is a predictor of oocyte competence in terms of a number of retrieved, mature and fertilized oocytes. The indicator can be used for prediction of clinical pregnancy rate after ICSI. It also gives information about the number of cleaved embryos and clinical pregnancy rate which needs to be further explored. 
